# Practical guide to statistical reporting in optical imaging

**DOI:** 10.1117/1.NPh.13.1.016601

**Published:** 2026-03-11

**Authors:** Xiaojun Cheng, Emily P. Stephen, Meryem A. Yücel

**Affiliations:** a Boston University, Neurophotonics Center, Biomedical Engineering Department, Boston, Massachusetts, United States; b Boston University, Department of Mathematics and Statistics, Boston, Massachusetts, United States

**Keywords:** statistical reporting, optical imaging, precision

## Abstract

Clear and consistent reporting of statistical values such as means, standard deviations, confidence intervals, p values, correlations, and test statistics is essential for reproducibility in neurophotonics. However, inconsistent rounding and unclear formatting remain common issues, particularly among new students and researchers. This technical note presents a concise and practical procedure for reporting statistical results with appropriate precision. It includes step-by-step rules, examples, and a summary table. The guide is intended as a quick reference for students, authors, and reviewers working with optical imaging data.

## 1 Introduction

Accurate statistical reporting is essential for ensuring that results are interpretable, reproducible, and meaningful.^1^^–^^4^ In neurophotonics, measurement variability arises from both instrumental and biological sources,^4^^–^^8^ making clear communication of uncertainty especially important. Confidence intervals (CIs), interquartile ranges (IQRs), and standard deviations (SDs) are among the most informative ways to express uncertainty. However, these statistics require appropriate precision to avoid implying false accuracy.^9^^–^^11^ This technical report addresses a common and often overlooked question: How many digits should be kept when reporting key statistical parameters? We provide a step-by-step procedure that aligns reported precision with the reliability of the estimates, allowing researchers to communicate results with clarity and integrity.

## 2 Step-by-Step Guide to Reporting Significant Digits for Means, Standard Deviations, and Confidence Intervals

Below, we propose a simple procedure to determine how many significant digits to retain when reporting.

Note: No rounding should be applied during preprocessing or analysis, as this can accumulate computational inaccuracies. Rounding is only performed at the final reporting stage for clarity.

Step 1. **Calculate the standard error (SE)**. The SE or the standard error of the mean is estimated as SE=σ^/N, where σ^ is the estimate of the standard deviation σ which is calculated from N independent measurements or samples. Please note that this equation applies specifically to the arithmetic mean value of a variable, which is the most common use case in optical imaging. For other statistics, such as median or proportions, the SE should be computed using the appropriate equation.^12^
Step 2. **Round SE**. Round the SE to one significant digit in most cases. Use two significant digits only when the first significant digit is 1 because rounding a value such as 0.01516 to one digit (0.02) introduces a large relative error. In this case, keeping two digits (0.015) provides a more accurate representation of the uncertainty.
Step 3. **Round mean, SD, and CI to match the decimal places of SE**. Round the mean, SD, and CI so that their decimal places match the decimal place implied by the rounded SE, because the SE determines the meaningful precision of the estimates.^13^^–^^15^ For example, if the SE is 0.3 (implying one decimal place of precision), the raw estimates (mean = 1.56, SD = 1.24, and 95% CI: 0.97 to 2.15) must all be rounded to one decimal place. Applying this rule yields the following: mean = 1.5, SD = 1.2, and 95% CI: 1.0 to 2.2.
Step 4. **Specifying the reported parameter**. Always specify whether you are reporting mean (SD) or a confidence interval (e.g., 95% CI). For example, “back-scattered light energy was 0.56 (0.02) mW, mean (SD)” or “cerebral blood-flow change: mean = 12%, 95% CI: 8 to 16%.”
  The SD describes variability within a dataset, indicating how individual observations differ from the mean and should be used to represent data dispersion. For expressing uncertainty in the estimated mean, 95% CIs are preferred over standard errors. A 95% CI provides an interpretable range of plausible values for the true population mean by combining the point estimate with its associated uncertainty on the same measurement scale. In contrast, standard errors quantify sampling variability without specifying a confidence level and can therefore be misinterpreted when reported in isolation.^1^^,^^16^ For these reasons, we recommend reporting 95% CIs when the goal is to communicate uncertainty about an estimated parameter.
  Special cases: Although the precision of reported values is determined by the SE, researchers are usually more interested in interpretability than in the instrument’s raw precision. When the measurement precision exceeds what is needed for interpretation, rounding the mean, SD, and CI to two significant digits is generally sufficient, provided their decimal places are consistent. Higher precision should be retained only when it is scientifically meaningful in a given field. For example, a digital scale may measure body weight to 0.0001 kg, but reporting a human participant’s weight as 52.1034 kg adds no practical interpretive value. In contrast, fields such as high-precision chemistry or physics may require reporting SDs with more digits if even tiny variations are meaningful.
  If the SD is similar to or larger than the mean, for example, mean (SD) = 345.6 (223.4), avoid rounding to coarse values with trailing zeros such as 300. This can misrepresent both central tendency and variability. Instead, keep two significant digits and use scientific notation if helpful, for example, mean $(\mathrm{SD})=3.5\times 10^{2}$ ($1.2\times 10^{2}$).
Step 5. **Report your numbers clearly and consistently**. We recommend reporting values as mean (SD) for descriptive statistics or mean (95% CI) when describing uncertainty about an estimated parameter. This notation avoids ambiguity and aligns with current reporting guidelines. If the “±” format is used, it should be clearly defined at first use (e.g., “95% CI: 12±4%”) and applied consistently throughout the paper.^1^ Avoid mixing formats within a paper and apply the same convention consistently across all results and figures.

## 3 Reporting Non-Normally Distributed Data

The reporting recommendations above apply primarily to approximately normal, unimodal distributions, for which the mean and SD provide meaningful summaries of central tendency and variability. When data are skewed, heavy-tailed, or otherwise non-normally distributed, the median and IQR should be reported instead. As with mean-based statistics, the reported precision of the median and IQR should reflect the measurement resolution and sample size, and excessive digits should be avoided. Results may be reported in the format median (IQR), with consistent rounding applied across all reported values. For example, if the distribution of the measured quantity were skewed, results could be reported as median (IQR) = 0.32 (0.21 to 0.44), using the same rounding principles described above.

## 4 Other Statistical Parameters

Researchers also commonly report p-values, correlation coefficients, and effect sizes; consistent formatting of these values aids accurate interpretation and comparison across studies.

p-values: Always specify the test statistic used to calculate the p-value (e.g., t-statistic, z-score, and F-value). Report p-values using two significant digits as the general rule. In cases where using two significant digits would require more than three decimal places, such as p=0.0017, round the value to three decimals instead, for example, p=0.002. For any p-value below 0.001, report it as p<0.001 to avoid implying false precision. If a journal asks for exact values for very small p-values, scientific notation with two significant digits may be used, for example, $p=2.3\times 10^{-5}$.

Multiple comparisons and corrected p-values: Statistical analyses in optical imaging and neuroimaging often involve multiple tests, for example, across channels, regions, or time points. As the number of statistical tests increases, the probability of false-positive findings also increases. When multiple comparisons are performed, corrected p-values should therefore be reported.

The authors should explicitly state whether and how multiple-comparison correction was applied and report adjusted values accordingly (e.g., false-discovery-rate-adjusted q-values or family-wise error-corrected p-values). For the corrected p-values, the same formatting and rounding rules described above for p-values should be applied. Uncorrected p-values may be reported for completeness when appropriate but should be clearly labeled as such.

t-values and F-values: Report with two decimal places and include degrees of freedom where applicable [e.g., t(21)=2.34, F(2,18)=5.12].

Correlation coefficients (r): Report with two decimal places (e.g., r=0.42), which is sufficient for interpretation.

Effect sizes (e.g., Cohen’s d and partial eta squared): Report with two decimal places to facilitate interpretation of practical significance (Cohen’s d=0.56).

## 5 Examples in the Field of Optical Imaging

To illustrate the practical application of these statistical reporting rules, we present two representative examples from neurophotonics research. These examples demonstrate how to calculate, round, and report mean, SD, and SE values clearly and consistently. Following the examples, Table 1 summarizes the recommended reporting formats for commonly used statistical parameters in optical imaging.

**Table 1:** Recommended statistical reporting formats for optical imaging.

Statistic	Recommended format	Recommended example	Not recommended example
Mean and SD	Mean and SD rounded to SE’s first significant digit’s decimal place	Mean (SD) = 0.56 (0.22) when SE = 0.05	Mean (SD) = 0.5643 (0.2232) when SE = 0.05
CIs	Match decimal precision to that of the reported estimate	Mean = 0.23, 95% CI: 0.12 to 0.35 or 95% CI: 0.23±0.11	Mean = 0.23, 95% CI: 0.12345 to 0.34567
p-values	Report p-values using two significant digits. When two significant digits would require more than three decimal places, round to three decimals instead	p<0.001 or $p=3.4\times 10^{-8}$	p=0.000000034
	For values below 0.001, report p<0.001. If exact reporting is required for very small p-values, use scientific notation		
t-values and F-values	2 decimal places, including degrees of freedom in parentheses when applicable	t(21)=2.34	t=2.3413492
Correlation coefficients (r)	2 decimal places	r=0.42	r=0.423765
Effect sizes (e.g., Cohen’s d and $\eta^{2}$)	Two decimal places	Cohen’s d=0.56	Cohen’s d=0.56382

Example 1: Researchers evaluated the average change in oxygenated hemoglobin (HbO) in the dorsolateral prefrontal cortex (DLPFC) during a two-back working memory task. Data were collected from 20 participants.

Raw values (before rounding): The mean ${\mathrm{HbO}}_{2}$ change was 0.3452  μM, with an SD of 0.1426  μM, based on a sample size (N) of 20.

Step 1. Calculate the standard error (SE): The SE is calculated as the SD divided by the square root of N, which gives
  SE=SD÷√N=0.1426÷√20≈0.1426÷4.4721≈0.0319.
Step 2. Round the SE: As the leading digit of the SE is “3,” keep one significant digit and round the SE to 0.03.
Step 3. Round the mean and SD to match the decimal places of the SE: Because the SE has two decimal places, round the mean and SD accordingly. The mean is rounded from 0.3452 to 0.35, and the SD is rounded from 0.1426 to 0.14.
Step 4. Report the results clearly: The HbO concentration increased by 0.35 (0.14)  μM, mean (SD), during the working memory task.

Test statistic reporting: Suppose a paired t-test comparing pre-task and task HbO levels yields: t=3.21, p=0.0046: Report with rounded off p-value: “Paired t(19)=3.21, p=0.005,” where 19 is the degrees of freedom, calculated as N−1.

Final reported result: “During the two-back working memory task, HbO concentration in the DLPFC increased significantly by 0.35 (0.14)  μM [mean (SD), N=20, paired t-test, one-sided: t(19)=3.21, p=0.005].”

Note: Please note that, if the distribution of HbO changes were skewed, results could be reported as median (IQR) = 0.32 (0.21 to 0.44) μM, using the same rounding principles described above.

Example 2: A neuroscientist is measuring wide-field fluorescence intensity using a camera. After measuring 10 independent samples, the average fluorescence intensity was 4.2356 ADU (arbitrary units), with an SD of 0.879 and SE of $0.879/\sqrt{10}=0.278$. Reporting the mean to four decimal places might seem impressive, but it can give a false impression about the precision. SE indicates uncertainty around the first decimal place, so it is more accurate and clearer to present the results as 4.2 (0.9) using mean (SD), to represent the sample’s variability. When the goal is to describe uncertainty in the mean estimate, report a confidence interval instead, for example, 4.2 (95% CI: 3.8 to 4.6). This approach provides a realistic sense of the measurement’s precision without distracting readers with insignificant digits.

## 6 Conclusion

Consistent and transparent statistical reporting supports the integrity and reproducibility of optical imaging research. By aligning the precision of reported values with the reliability of their estimates, particularly through rounding based on the SE, researchers can communicate results more clearly and avoid false impressions of accuracy. This practical guide provides straightforward rules and examples that help standardize reporting practices, benefiting authors, reviewers, and readers alike. Adopting these guidelines will foster clearer interpretation, reduce miscommunication, and ultimately strengthen scientific rigor within the neurophotonics community.

## Data Availability

No new data were created or analyzed in this article. Data sharing is not applicable.
